# Suicidal Ideation in a Population-Based Sample of Adolescents: Implications for Family Medicine Practice

**DOI:** 10.5402/2013/282378

**Published:** 2013-01-30

**Authors:** Esme Fuller-Thomson, Gail P. Hamelin, Stephen J. R. Granger

**Affiliations:** ^1^Factor-Inwentash Faculty of Social Work and Department of Family and Community Medicine, University of Toronto, 246 Bloor Street West, Toronto, ON, Canada M5S 1A1; ^2^Kinark Child and Family Services, 34 Simcoe Street, Suite 301, Barrie, ON, Canada L4N 6T4; ^3^Parent and Child Capacity Building Team, Peel Children's Aid Society, 6860 Century Avenue, Mississauga, ON, Canada L5N 2W5

## Abstract

*Introduction*. This study investigated the relationship between suicidal ideation and demographic characteristics, health conditions, depression, and health care utilization patterns among adolescents. *Methods*. Secondary analysis of the regionally representative Canadian Community Health Survey conducted in 2000/2001 (response rate 85%). Adolescents aged 15 to 19 who reported suicidal ideation in the previous year (*n* = 260) were compared with their peers who did not (*n* = 5528). The association between suicidal ideation and socio-demographic and health characteristics were investigated. *Findings*. Almost three-quarters (73%) of suicidal adolescents had not spoken with any health professional about mental health issues in the preceding year. Despite the fact that 80% of suicidal adolescents had regular contact with their family doctor, only 5% had consulted with them about mental health issues. In addition to the well-known risk factors of depression and stress, suicidal ideation was highly elevated in adolescents with two or more chronic health conditions, self-reported poor health, migraines, and back pain and those whose activities were prevented by pain (*P* < .05). Other characteristics significantly correlated with suicidal ideation included smoking, living in single parent families, and having lower levels of social support. *Conclusions*. Family physicians should regularly screen for suicidal thoughts in their adolescent patients with these characteristics.

## 1. Introduction

The loss of a patient, particularly an adolescent patient, to suicide is one of the most distressing events a clinician can face [1]. It is imperative that family doctors possess knowledge and understanding of the characteristics of suicidal adolescents in order to more accurately identify and intervene at an early stage. There is a significant gap in services for youth with all types of mental health issues. A recent Canadian Study showed half of suicidal adolescents had not accessed any mental health services [2]. Often the only connection these vulnerable youth have with the health care system is their family doctor.

Suicide is the third leading cause of death for teenagers in the United States [3] and the second leading cause of death for Canadian adolescents [4]. Suicide ideation and attempts are known risk factors for suicide completion [5–10], with an estimated 15 to 20 attempted suicides to every completed suicide [10]. Researchers note that the overall low base rate of completed suicide for youth makes it very difficult, even with a good screening tool, to predict the level of risk for completed suicide and for this reason they suggest concentrating on the phenomena associated with suicidal ideation in youth [11–13]. The prevalence of suicidal thoughts among adolescents is quite high, with an estimated 20–30% of adolescents reporting them at some point [14].

The rates at which youth present with suicidal ideation, suicide attempts, and suicide are alarmingly stable over the past twenty years. There has been extensive ongoing research [6–10, 15, 16] into the risk factors and treatments for suicidal ideation and behavior. A systematic review of the literature concluded that there was strong evidence that the following factors are associated with adolescent suicidal phenomena: depression, alcohol abuse, use of hard street drugs, suicidal behavior among friends, living apart from parents, family conflict, unsupportive parents, and a history of abuse [17]. Less conclusive but promising evidence also suggests that poor physical health, sexual activity, and physical disability are associated with suicide attempts [17]. Unfortunately, there are few representative community studies discussing the use of family doctors and the use of mental health services among adolescents with suicidal ideation.

## 2. Material and Methods

### 2.1. Data Source

This study investigated the relationship between suicidal ideation and demographic characteristics, health conditions, psychological characteristics and health care utilization patterns among a regionally representative sample Canadians aged 15 to 19. We also report utilization patterns of health and mental health services by Canadian adolescents with suicidal ideation. The analyses reported in this paper used the public use data files of the Canadian Community Health Survey (CCHS) conducted in 2000/2001. The target population of the CCHS covered approximately 98% of the Canadian population aged 12 or older. The CCHS had a response rate of 84.7% after 14 months of collection, which resulted in a final sample of 130,880 respondents [18]. The survey was administrated through in-home face-to-face interviews with 88% of the sample and telephone surveys conducted through home phone numbers for the remaining 12% of the sample.

Only six provinces (Nova Scotia, New Brunswick, Quebec, Ontario, Manitoba, Alberta) and one territory (Yukon) opted for the inclusion of questions on suicidal ideation. Thus, of the original 11,081 respondents aged 15 to 19 in the CCHS, only 5,788 were asked questions on suicidal ideation. These 5,788 respondents make up the sample for subsequent analyses. In the bivariate tables, we present data on several characteristics which were only asked in a sub-sample of the above provinces. Thus, self-report of life stress, hours per week watching television, whether the adolescent had ever had sexual intercourse, and levels of social support have smaller sample sizes than the other characteristics reported. These questions, in addition to income level, which had a substantial amount of missing data, were therefore excluded from the logistic regression analyses. The logistic regression analyses included 5,471 respondents who had complete data on all the variables in the analysis.

### 2.2. Statistical Analyses Strategy

 Adolescents who reported suicidal ideation in the previous year (*n* = 260) were compared with their peers who did not (*n* = 5528). The frequency of different forms of health care utilization was reported for adolescents who had suicidal ideation. Chi-square analyses were conducted for categorical variables and independent *t*-tests were conducted for interval and ratio level variables comparing teens with and without suicidal ideation. All data were weighted to adjust for probability of selection. Two logistic regression analyses of suicidal ideation were conducted. The first analysis included 5 demographic characteristics (gender, school attendance, race, living arrangement and whether the adolescent reported they sometimes or often did not have enough food to eat due to lack of money), 2 health behaviors (smoking status and alcohol dependency) and 6 physical health characteristics (self-perceived health status, whether their activities were prevented by pain, number of chronic conditions and whether the teenager had asthma, back problems or migraines). The second logistic regression included all of the above variables and depression.

### 2.3. Measures

#### 2.3.1. Outcome Variable: Suicidal Ideation

Respondents in the CCHS were asked two questions on suicidal ideation: “Have you ever seriously considered committing suicide or taking your own life.” Those who said yes were asked, “Has this happened in the past 12 months?” Therefore, our suicidal ideation variable was dichotomized into considered suicide in the past 12 months versus not.

#### 2.3.2. Demographic Characteristics

In the bivariate analyses, the following demographic variables were investigated: (1) gender; (2) educational enrolment status (currently attending school/college or university versus not); (3) race (self-report: white versus non-white); (4) living arrangement (adolescent lives with two parents, with one parent, unattached, other). The “other” category includes living with a spouse and/or a child. The household's financial situation was measured in several ways: (5) Total household income. (6) Income adequacy (low, medium, high) was calculated based upon total household income and number of people in the household. (7) Extreme poverty was assessed through the question, “In the past 12 months, how often did you or anyone else in your household not have enough food to eat because of a lack of money?” (never versus sometimes or often).

#### 2.3.3. Risk Behaviors

In several of the regions, respondents were also asked if they had ever had sexual intercourse. Smoking status identified those who had never smoked in comparison to those who were former or current smokers. Alcohol dependency was based on questions from the Composite International Diagnostic Interview-Short Form (CIDI-SF) developed by Kessler et al. [19]. This scale has been shown to have high sensitivity and specificity [19]. If a teenager responded that they had 5 or more drinks at least once a month during the last 12 months, they were asked a series of questions about symptoms of alcohol dependence (e.g., being drunk or hungover while at work or school or while taking care of children; experiencing a persistent desire for alcohol). If they reported at least three of these behaviors in the past month, they were classified as “alcohol dependent” [20].

#### 2.3.4. Physical Health Characteristics

 Self-reported health status was derived from the question “In general, would you say your health is excellent, very good, good, fair, poor.” The responses were dichotomized into excellent, very good or good, versus fair or poor. The survey participants were asked if they were usually free of pain and discomfort. If they responded no, they were asked how many activities their pain or discomfort prevented (none, a few, some, most). The answers were then dichotomized into two categories (usually no pain or discomfort or pain prevents no activities versus pain prevents few activities, some activities or most activities). The number of chronic conditions among the CCHS respondents was calculated from summing the number of the following 17 conditions that had been diagnosed by a health professional and that had lasted six months or more: asthma, back problems, migraine headaches, arthritis or rheumatism, fibromyalgia, inflammatory bowel disease, cataracts, chronic bronchitis, emphysema or chronic obstructive pulmonary disease, diabetes, epilepsy, heart disease, cancer, stomach or intestinal ulcers, effects of a stroke, glaucoma, and chronic fatigue syndrome. These responses were summed into three categories (no chronic conditions, 1 condition, 2 or more conditions). Only the first three problems were experienced by more than 5% of 15–19 year old respondents, and thus further analysis explored the association between each of these conditions and suicidal ideation. 

#### 2.3.5. Mental Health Characteristics

Those aged 18 and over in several of the provinces were asked about their stress level: “Thinking about the amount of stress in your life, would you say that most days are (not at all stressful, not very stressful, a bit stressful, quite a bit stressful, extremely stressful)?” The answers were dichotomized with the first three categories classified as “not stressful” and the latter two classified as “stressful”. 

Respondents were diagnosed as depressed if they had a probability of depression of 90% or greater as classified by Kessler and Mroczek's scale based on a subset of 21 items from the Composite International Diagnostic Interview (CIDI). The questions cover a cluster of symptoms for depressive disorder, which are listed in the 1987 Diagnostic and Statistical Manual of Mental Disorders of the American Psychiatric Association. This short-form (SF) scale was developed to operationalize Criteria A through C of the DSM-III-R diagnosis of major depressive episode. The sensitivity of the CIDI-SF was 89.6% and specificity was 93.9% with the total classification accuracy 93.2% for a major depressive episode in comparison to CIDI.

#### 2.3.6. Characteristics of Social Support and Excessive Television Viewing

In some of the provinces, four types of social support were evaluated using the Medical Outcomes Study Social Support Survey: Emotional and informational support (e.g., empathic understanding and guidance), tangible support (e.g., material or behavioural assistance), positive social interaction (e.g., doing fun things together), and affection (e.g., love and affection). This scale had excellent reliability and validity [21]. We also investigated the association between suicidal ideation and excessive television viewing (defined as 20 or more hours per week watching television).

#### 2.3.7. Health Care Utilization

Use of a family doctor was determined through the following question: “Not counting when you were an overnight patient in a hospital, nursing home or convalescent home, in the past 12 months, how many times have you seen, or talked on the telephone, about your physical, emotional or mental health with a family doctor or general practitioner?” Responses were dichotomized into never/ever. 

Respondents' contact with mental health care professionals was determined by the question: “In the past 12 months, have you seen or talked on the telephone to a health professional about your emotional or mental health?” Those who answered yes were asked how many times they visited a mental health professional (coded as no visits, 1–3 visits, 4 or more visits). Respondents were then asked “Whom did you see or talk to?” Family Doctor was one of several response options.

## 3. Results

 As shown in Table 1, females had higher rates of suicidal ideation than males (4.7% versus 2.9%; *P* < .001). Only significant findings with *P* values less than .05 are reported. Suicidal ideation was significantly more prevalent among those not attending school or college, unattached individuals and those living in single parent families as opposed to those living with two parents, and those who reported that they did not always have enough food to eat due to lack of money. The average household income of suicidal teenagers was significantly lower than that of their nonsuicidal peers. Each of the health risk behaviors (having had sexual intercourse, type of smoker, and alcohol dependency) was associated with an elevated prevalence of suicidal ideation. The prevalence of suicidal thoughts was higher among teens who reported poorer health status, those who had two or more chronic conditions, particularly those with migraine headaches and/or back problems, those whose activities were prevented by pain, and those who reported high levels of stress. Teenagers who were depressed had approximately ten times higher prevalence of suicidal ideation than the nondepressed (23.1% versus 2.1%). In comparison to adolescents without suicidal ideation, those who reported suicidal ideation had much lower levels of all four types of social support investigated. Teens who spent 20 or more hours a week watching television had almost double the prevalence of suicidal ideation in comparison to teens who did not watch as much television.

 The first logistic regression analysis (which did not include the variable on depression) indicated that women had 54% higher odds of suicidal ideation than men and that teens living in single parent households had 71% higher odds of suicidal ideation in comparison to those in two parent families (see Table 2, Model 1). Former or current smokers had five times the odds of reporting suicidal thoughts in comparison to teens who had never smoked. Adolescents who were alcohol dependent had more than twice the odds of suicidal ideation in comparison to their peers without alcohol dependency. Teens whose activities were prevented by pain had twice the odds of suicidal ideation in comparison to those without any activity limitation. According to the Nagelkerke R-square statistic, the variables in the first model explained 12.7% of the variability in suicidal ideation, which increased to 22.9% when depression was added to the model (please see Table 2, Model 2). Those who were depressed had nine times the odds of reporting that they had suicidal thoughts in the preceding year when compared with their nondepressed peers. Once depression was included in the model, only two other characteristics remained statistically significant: living in a single parent family and smoking status. 

Our univariate analyses of health care utilization indicated that suicidal adolescents were not getting the help they needed: almost three-quarters (73%) of suicidal adolescents had not spoken about mental health issues with *any* health professional (including family doctors, psychiatrists, psychologists and/or social workers). Only 18% of suicidal teens had four or more mental health visits with any health professional. Our particular interest was to focus on the use of family doctors by teens with suicidal ideations. Although 80% of suicidal adolescents reported that they had seen or spoken on the phone with their family doctor in the preceding year about any issue, only 5% indicated that they had consulted with their family doctor about a mental health issue.

## 4. Discussion 

 The vast majority of suicidal teens were not receiving any mental health services from health professionals. Our finding that only 27% of suicidal adolescents had consulted with any mental health professional in the past year is comparable to Wilson's finding that only 1 in 4 adolescents with a mental health problem seek medical care to address the problem [12]. 

Four out of five suicidal teens had seen or talked on the phone with their family doctor in the past year, presenting an important opportunity to discuss mental health concerns. Apparently, the majority of adolescents with suicidal ideation did not present these issues: only one in twenty suicidal teens reported that they had spoken to their family doctor about mental health concerns. Although general practitioners (GP), such as family practitioners, are often the first point of contact for adolescents seeking health care, barriers to accessing mental health assistance from GPs include “limited knowledge about the types of help GPs provide; perceived difficulties in the doctor-patient relationship; fears about both the process and content of a GP consultation” [12, p. 345]; and difficulties accessing services [22]. Literature suggests that parents' and youth's perceptions of a mental health problem affect whether youth access mental health services [23]. Barriers to service use include beliefs that the mental health problem is not serious enough and a desire to solve the problem oneself [23]. When these barriers can be overcome, research has found that youths are often more comfortable discussing issues with their GP than with a specialist [24].

 Therefore, it appears that the health professional must take the initiative to investigate potential suicidality among their adolescent patients. A recent study concluded that GPs do not routinely ask adolescent patients about suicidal ideation and often lack time to properly screen patients [24]. Researchers have proposed that medical practitioners take individualized approaches with clients to focus on their unique strengths, vulnerabilities, and specific circumstances when assessing suicidality [25]. Improved knowledge about the characteristics associated with elevated risk of suicidal ideation could improve targeting and outreach efforts by family physicians.

 In identifying their most vulnerable teen patients, the findings of our population-based study indicate that doctors may benefit from considering suicidal risk factors in addition to the usual “red flags” of depression [17] and stress [26]. Of particular relevance to family physicians, suicidal ideation was significantly elevated in teens with back problems and/migraine headaches, two or more chronic conditions, and poor self-reported health. Other studies have identified chronic illness as a risk factor for adolescent suicidal ideation [27, 28], particularly when comorbid with depression [29]. In a prospective study [30], migraines have been found to be an independent factor associated with suicide attempts, even in the absence of a major depressive disorder. Our finding that those whose activities were prevented by pain had more than triple the prevalence of suicidal ideation in comparison to those without activity limitations (12.6% versus 3.5%) builds on previous research showing positive associations between suicidal ideation and pain intensity [31] and chronic pain [32, 33], although the link between suicidality and both back pain and activity limitation due to pain have not been as extensively studied in the adolescent population.

Additionally, women, teens from single parent families, those without adequate food due to financial problems, smokers, and those who were sexually active had a high prevalence of suicidal ideation as did those with low levels of social support. Family physicians could target adolescent patients with these mental health, health, and sociodemographic characteristics for sensitive assessment of suicidal risk as an important first step in improving services to these vulnerable and underserved adolescents. 

In keeping with the previous research, we found female adolescents had much higher risk of suicidal thoughts than their male peers. The risk ratio in this study was 1.62, very similar to that found in the average of 5 previous studies on the phenomenon (1.58, 95% C.I. 1.50 to 1.67) [14]. While females have higher risks of suicidal thoughts, plans, and attempts, it is, in fact, adolescent males who have much higher rates of suicide completion [34]. 

Our findings that 2.9% of males aged 15 to 19 and 4.7% of female adolescents reported suicidal thoughts in the previous year was well below the average found in other studies (19.3%, 95% C.I. 11.7% to 27% at some point in a 12 month period) [35]. There are several possible reasons for this: interview surveys (such as the CCHS) typically report much lower rates of all suicidal phenomena than anonymous questionnaires [35]. Furthermore, the vast majority of the previous studies had been school based, not community based [35]. It is probable that adolescents are less likely to report suicidal thoughts when interviewed in their home environment than in a school setting.

We found a positive correlation between a number of health risk behaviors and suicidal ideation which had also been identified in other studies. These factors include smoking [36, 37], sexual activity and alcohol dependency. Evidence suggests that smoking mediates other psychosocial causes of suicidal ideation (e.g., depression) and is likely not a causal pathway [37]. Other researchers have also shown that among adolescent females, sexual activity and sexual risk-taking is associated with increased suicidal ideation and suicide attempts [36, 38–43]. Both male and female adolescents who used alcohol at the time of sexual intercourse were found to have greater odds of considering a suicide attempt [36]. Within the adolescent population, sexual risk-taking behavior has been identified as a psychosocial and behavioral risk factor interrelated with suicidal ideation.

We found that not attending school was associated with much higher rates of suicidal ideation, a finding that replicated earlier research [44]. Depression, however, is also highly correlated with school drop-out and thus it is unclear whether the relationship is direct or indirect. Living arrangement was also a significant factor. Previous research [45] supports our finding that those who lived with neither parent had higher levels of suicidal ideation than those living in two parent families. However, our finding that adolescents from single parent families had higher suicidal ideation rates than those in two parent families is in keeping with some earlier research for example, [46], but in contrast to others (e.g., [45]).

We found that suicidal ideation was higher in those with lower household income, measured in dollars, and those with insufficient food due to economic problems. However, when income was divided into low, medium, and high categories, we did not find a significant association with suicidal ideation. The association between poverty and suicidal ideation may result from the increased stress and higher level of hopelessness among those in extreme financial need [47, 48]. Other studies suggest that it is particularly food insufficiency, and not low income levels in general, which is strongly correlated to dysthymia, suicidal ideation, and suicide attempts [49]. 

Evidence suggests that individuals often use entertainment media when experiencing stress and anxiety [50], and when feeling lonely [51]. In their nationally representative study, Bickham and Rich [52] found that children who spent more time watching violent television alone spent less non-television time with friends. Our study indicated a significantly higher prevalence of suicidal ideation among those watching television more than 20 hours per week. Our data do not allow us to know if those with excessive television viewing were socially isolated or lonely. In future research on adolescents and suicidality, attention to high levels of media exposure and its potential link to social isolation would be helpful. Research suggests the importance of understanding the role of social integration and isolation with suicidal behaviour [53]. A number of studies have found social isolation to be associated with increased risk of suicide attempts [54] and suicidal ideation [55, 56].

Adolescents in the present study who reported suicidal ideation had low levels of all four types of social support. This finding supports previous studies that have found social support to be a protective factor against suicidality [57–59], particularly perceived parental support [57, 58]. 

In our study, 10.2% of the variability in suicidal ideation was explained by depression, even after a wide range of other risk factors had been included in the analysis. Other researchers have also emphasized that presence of a depressive mood disorder is among the most powerful predictors of suicidal ideation and suicide attempt [60]. Further elevations in the risk for suicidal behavior occurs when depression or affective disorders are present in combination with substance abuse [61–64]. However, it is positive to note that three quarters of the depressed respondents were not suicidal. Future research efforts could focus on reasons why some depressed adolescents are suicidal while the majority are not in order to inform strategies to decrease the prevalence of suicidal ideation. A major limitation of our study was the lack of data in the CCHS on several important risk factors for suicidal ideation including homosexuality and bisexuality [65] and whether the teen had a peer or family member who had attempted suicide [17].

## 5. Conclusion 

 Family doctors are suicidal adolescents' major point of contact with the health care system. Although four out of five suicidal teens had met with a family physician in the year preceding the survey, only one in twenty had discussed any mental health concerns with their family doctor. Clearly, the onus is on the family doctor to initiate discussions related to depression and suicidal thoughts because teenagers rarely disclose these issues to their doctors without prompting. This study provides a profile of teens most at risk for suicidal ideation, which should improve targeting and outreach to the most vulnerable. In addition to the well-known risk factors of depression and stress, suicidal ideation was highly elevated in adolescents with two or more chronic health conditions, self-reported poor health, migraines, and those whose activities were prevented by pain. Teens with these conditions are likely to be heavier users of family medicine services. Other characteristics significantly correlated with suicidal ideation include lower income, smoking, living alone or in single parent families, having lower levels of social support, and watching excessive amounts of television. Family physicians should regularly screen for suicidal thoughts in their adolescent patients with these characteristics in order to intervene as early as possible.

## Figures and Tables

**Table 1:** Bivariate analysis of suicidal ideation in 12 months preceding the survey (*n* = 5,788). Source: Canadian Community Health Survey 1.1 [18].

Variable	Total (*n*)	% Suicide ideation or means (SD)	*P* value
Demographic characteristics			

Gender			
Male	2877	2.9%	<.001
Female	2911	4.7%	
Currently attending school/college			
Yes	4480	3.3%	<.001
No	1308	5.9%	
Race (Self-report)			
Non-white	691	2.8%	.07
White	5006	4.0%	
Living arrangement			
Child with 2 parents	3781	2.9%	<.001
Child with single parent	713	6.0%	
Unattached	448	6.5%	
Other	767	4.6%	
Total household income mean (S.D)			
Not suicidal	4716	$55,860 ($27,210)	<.001
Suicidal	222	$48,780 ($26,175)	
Income adequacy			
Low	668	5.1%	.12
Middle	1197	3.8%	
High	3094	3.4%	
Not enough to eat due to lack of money			
Always enough food	5234	3.7%	.01
Sometimes/often not enough	504	6.1%	

Risk behaviours			

Had sexual intercourse			
Yes	821	7.1%	<.001
No	1197	2.1%	
Type of smoker			
Never smoked	2780	1.2%	<.001
Former or current smoker	2978	6.9%	
Alcohol dependency			
Not dependent	5440	3.5%	<.001
Dependent	288	13.6%	

Physical health			

Self-perceived health			
Excellent/good health	5497	3.6%	<.001
Fair/poor health	291	8.7%	
Activities prevented by pain			
No activities prevented	5567	3.5%	<.001
Some activities prevented by pain	216	12.6%	
Number of chronic conditions			
No conditions	4216	3.3%	<.001
1 condition	1189	3.8%	
2 or more conditions	376	10.6%	
Has Asthma			
No	5058	3.7%	.16
Yes	729	4.8%	
Has back problems			
No	5306	3.5%	<.001
Yes	482	7.6%	
Has migraine headaches			
No	5363	3.6%	<.001
Yes	425	7.2%	

Mental health			

Life stress			
Not at all/a bit	1874	3.2%	.02
Quite a bit/extremely	403	5.5%	
Depression			
No	5257	2.1%	<.001
Yes	512	23.1%	

Social support			

Emotional/informational support mean (S.D.)			
Nonsuicidal	3910	28.1 (4.9)	<.001
Suicidal	182	24.7 (7.2)	
Positive social interaction Mean (S.D.)			
Nonsuicidal	3917	14.4 (2.3)	<.001
Suicidal	183	13.3 (2.9)	
Affection mean (S.D.)			
Nonsuicidal	3918	10.7 (1.9)	<.001
Suicidal	184	9.4 (3.0)	
Tangible social support Mean (S.D.)			
Nonsuicidal	3922	14.3 (2.4)	<.001
Suicidal	184	13.0 (3.2)	
Hours/week watching TV			
0–20 hours	2380	3.4%	<.01
>20 hours	485	6.3%	

**Table 2:** Logistic regression analysis of suicidal ideation in 12 months preceding the survey (*n* = 5,471). Source: Canadian Community Health Survey 1.1 [18].

Variable	Model 1: Odds ratio depression excluded	95% Confidence Interval	Model 2: Odds ratio depression included	95% Confidence Interval
Demographic characteristics				

Gender				
Male	**1.00**	**Reference**	1.00	Reference
Female	**1.54**	**(1.15, 2.08)**	1.07	(0.78, 1.47)
Currently attending school/college				
Yes	1.00	Reference	1.00	Reference
No	1.18	(0.85, 1.64)	1.17	(0.83, 1.64)
Race (Self-report)				
Non-white	1.00	Reference	1.00	Reference
White	1.02	(0.66, 1.59)	1.09	(0.69, 1.73)
Living arrangement				
Child with 2 parents	1.00	Reference	1.00	Reference
Child with single parent	**1.71**	**(1.18, 2.46)**	**1.70**	**(1.16, 2.48)**
Unattached	1.37	(0.85, 2.21)	1.20	(0.73, 1.99)
Other	1.01	(0.66, 1.54)	1.06	(0.68, 1.66)
Not enough to eat due to lack of money				
Always enough food	1.00	Reference	1.00	Reference
Sometimes/often not enough	1.15	(0.74, 1.77)	0.82	(0.51, 1.31)

Risk behaviours				

Smoking status				
Never smoked	1.00	Reference	1.00	Reference
Former or current smoker	**5.03**	**(3.41, 7.40)**	**4.21**	**(2.84, 6.23)**
Alcohol dependency				
Not dependent	1.00	Reference	1.00	Reference
Dependent	**2.27**	**(1.43, 3.56)**	1.48	(0.90, 2.43)

Physical health				

Self-perceived health				
Excellent/good health	1.00	Reference	1.00	Reference
Fair/poor health	1.53	(0.94, 2.50)	1.30	(0.77, 2.19)
Activities prevented by pain				
No activities prevented	1.00	Reference	1.00	Reference
Some activities prevented by pain	**1.99**	**(1.18, 3.36)**	1.64	(0.93, 2.90)
Number of chronic conditions				
No conditions	1.00	Reference	1.00	Reference
1 condition	0.84	(0.48, 1.49)	0.83	(0.46, 1.48)
2 or more conditions	1.34	(0.50, 3.56)	1.06	(0.37, 3.05)
Has asthma				
No	1.00	Reference	1.00	Reference
Yes	1.05	(0.57, 1.94)	0.95	(0.50, 1.82)
Has back problems				
No	1.00	Reference	1.00	Reference
Yes	1.28	(0.67, 2.45)	1.31	(0.66, 2.60)
Has migraine headaches				
No	1.00	Reference	1.00	Reference
Yes	1.35	(0.71, 2.56)	1.25	(0.64, 2.45)

Mental health				

Depression				
No	—	—	1.00	Reference
Yes			**8.97**	**(6.49, 12.40)**

Model characteristics				

Chi-Square of the model	197.7		360.1	
*P* value of the model	*P* < .001		*P* < .001	
Nagelkerke Pseudo *R*-Square	.127		.229	
